# Use of a Smartphone App for Weight Loss Versus a Paper-Based Dietary Diary in Overweight Adults: Randomized Controlled Trial

**DOI:** 10.2196/14013

**Published:** 2020-07-31

**Authors:** Jeong Sun Ahn, Heejin Lee, Jiae Kim, Haemin Park, Dong Woo Kim, Jung Eun Lee

**Affiliations:** 1 Department of Food and Nutrition College of Human Ecology Seoul National University Seoul Republic of Korea; 2 Platform Development Team Bluecore Seoul Republic of Korea; 3 Department of Home Economics Korea National Open University Seoul Republic of Korea

**Keywords:** smartphone app, mobile phone, dietary self-monitoring, randomized controlled trial, weight loss

## Abstract

**Background:**

Mobile health (mHealth) tools may be useful platforms for dietary monitoring and assessment.

**Objective:**

This study aims to evaluate the effectiveness of a mobile dietary self-monitoring app for weight loss versus a paper-based diary among adults with a BMI of 23 kg/m^2^ or above.

**Methods:**

A total of 33 men and 17 women aged 18-39 years participated in a 6-week randomized controlled trial. We randomly assigned participants to one of two groups: (1) a smartphone app group (n=25) or (2) a paper-based diary group (n=25). The smartphone app group recorded foods and dietary supplements that they consumed and received immediate dietary feedback using Well-D, a dietary self-monitoring app developed by our team. The paper-based diary group was instructed to record foods or supplements that they consumed using a self-recorded diary. The primary outcomes were weight, BMI, waist circumference, body fat mass, and skeletal muscle mass. We also examined changes in nutrient intake, including energy, carbohydrate, protein, fat, dietary fiber, vitamins, and minerals, using 3-day 24-hour recalls. Differences in changes between the two groups were analyzed using independent t tests or Wilcoxon Mann-Whitney tests. All of the data were analyzed using intent-to-treat analysis.

**Results:**

The mean number of days recorded was 18.5 (SD 14.1) for the app group and 15.5 (SD 10.1) for the paper-based diary group. The differences in changes in weight, BMI, and waist circumference were not significantly different between the app group and paper-based diary group (*P*=.33, .34, and .70, respectively). Similarly, changes in body fat mass or skeletal muscle mass did not differ between the two groups (*P*=.71 and .054, respectively). Although energy intake was reduced in both groups, there was no significant difference in changes in energy intake between the two groups (*P*=.98).

**Conclusions:**

There were no differences in changes in anthropometric measures and nutrient intake between the app group and the paper-based diary group. Both mobile dietary self-monitoring app and paper-based diary may be useful for improving anthropometric measures.

**Trial Registration:**

Clinical Research Information Service KCT0003170; https://cris.nih.go.kr/cris/search/search_result_st01_en.jsp?seq=11642&ltype=&rtype=

## Introduction

Noncommunicable diseases (NCDs) were responsible for 71% of all deaths globally in 2016, and obesity is a risk factor for NCDs like diabetes, coronary heart disease, stroke, and cancer [1]. The World Health Organization (WHO) announced that one of the global NCD targets is to halt the rise of obesity [2]. Despite multifaceted efforts to prevent obesity, the prevalence of obesity has nearly tripled between 1975 and 2016 [3]. In Korea, the prevalence of a BMI 25 kg/m^2^ increased in men from 36.6% in 2008 to 44.7% in 2017 [4]. The prevalence of hypercholesterolemia doubled between 2008 and 2018 in both men and women, from 10.6% to 20.9% in men and from 11.8% to 21.4% in women [4]. Increasing obesity is partly due to changes in lifestyle factors, including eating energy-dense foods and foods high in fat and sugars as well as low physical activity [1]. Therefore, dietary intervention is a key strategy to reduce the obesity epidemic.

Dietary modification approaches for obesity management often involve multiple strategies from governments, businesses, communities, individuals, and families. Use of mobile devices, including smartphones, tablets, laptops, wearable devices, and barcode scanners, is thought to increase accessibility at a lower cost by reducing face-to-face, in-person education, clinic visits, and phone calls. Although several studies have shown that mobile interventions led to weight loss [5], a comparison of the effectiveness of mobile health (mHealth) tools for weight loss with conventional methods such as the paper-based diary is needed.

The third global survey on eHealth conducted by the WHO Global Observatory for eHealth defines mHealth as “the use of mobile devices, such as mobile phones, patient monitoring devices, Personal Digital Assistants (PDAs), and wireless devices, for medical and public health practice” [6]. There has been an increase in the number of mHealth solutions because information and communications technology (ICT) has become an integral part of daily life [7]. The WHO announced that 87% (n=109) of countries worldwide have at least one mHealth program in their country [7]. A systematic review of 24 intervention studies examining the potential of mobile apps for health and fitness reported high acceptability of smartphone apps for health behavior change [8].

Mobile technology may be feasible, sustainable, and cost-effective for weight loss. A systematic review of the literature on self-monitoring in weight loss showed that self-monitoring tools (eg, the paper diary, web tools, PDAs, and electronic digital scales) helped individuals lose weight [9]. That systematic review focused on self-monitoring strategies based on works published from 1993 to 2009; most of these studies used a paper diary and 5 used digital technology. A recent qualitative summary of 7 randomized clinical trials suggested that mobile technology interventions facilitated weight loss among individuals who were overweight and obese [10]. Several previous studies investigated the effectiveness of mHealth tools for weight loss or dietary behavior change by comparing them with traditional paper-based methods [11-15] and found that the use of mHealth tools was not superior to paper-based methods.

We have developed a mobile dietary self-monitoring app, Well-D, the features of which have been described elsewhere [16]. In brief, users can log foods or dietary supplements they consume through the Well-D app and receive real-time personalized dietary feedback. Key features of Well-D include sign-up and profile input, log-in, main page, logging meals, food data creation, recipe data creation, logging favorite foods, logging dietary supplements, supplement data creation, display of foods and supplements consumed, and dietary feedback and monitoring.

South Korea had the highest rate of smartphone ownership and internet usage worldwide in 2018, followed by Israel and the Netherlands [17]. Although mHealth has high potential to facilitate health management, health promotion, and disease prevention in South Korea, the effectiveness of morbidity self-management (eg, diabetes [18] and obesity with sleep apnea [19]) through smartphone use has been studied in only a few randomized controlled trials (RCTs). We conducted an RCT in South Korea to examine weight loss using a self-monitoring dietary app. This study aims to evaluate the effectiveness of the dietary self-monitoring app Well-D for weight change by comparing it with paper-based diary use.

## Methods

### Study Participants

We recruited participants between February 6, 2018, and April 12, 2018, via poster advertisement at Seoul National University and web-based announcements. The inclusion criteria were as follows: (1) 18-40 years of age, (2) BMI ≥23 kg/m^2^, (3) willingness for weight loss, and (4) smartphone ownership. We excluded participants if they were pregnant or lactating. This study was approved by the Seoul National University Institutional Review Board (IRB #1710/003-007). The trial was registered with the Clinical Research Information Service (KCT0003170).

### Screening and Randomization

A two-arm, parallel RCT was conducted. Potential participants contacted author JSA via phone to show their willingness to participate in the intervention study. Potential participants were invited to attend a baseline session held at Seoul National University (30-45 minutes). Before starting the baseline session, potential participants reported their age, and their height and weight were measured using a stadiometer to confirm eligibility. All eligible participants returned a written informed consent prior to enrollment. Participants received 20,000 KRW (approximately $17 USD) for attending each of the three visits.

**Figure 1:**
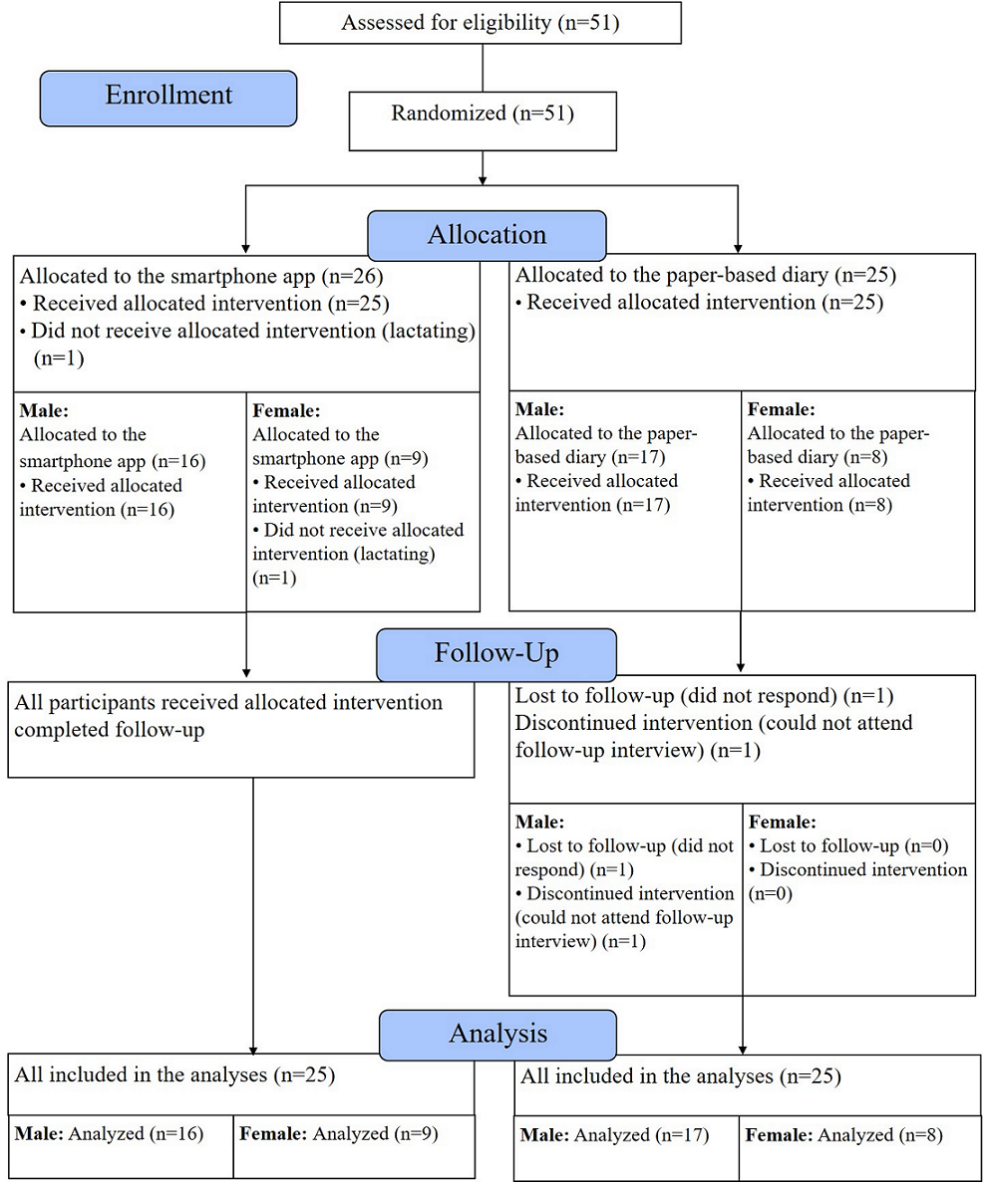
Flow diagram of participants.

Participants were randomly assigned with a 1:1 allocation to the Well-D app group and the paper-based diary group using a random number table generated by PROC PLAN in SAS version 9.4 (SAS Institute). The allocation sequence was concealed to both JSA and each participant, and the intervention arm allocation was sealed after the participant signed the consent form at the visit site. The participant was informed whether he or she was assigned to the intervention (smartphone group) or the control group (paper-based diary group). Figure 1 presents the flow diagram of inclusion of participants.

### Intervention

Both groups were instructed to record foods or supplements that they consumed during the 6-week intervention period. The energy goal was reducing 500 kcal/day from the the estimated energy requirement (EER) in both groups. Participants in the app group received a link to download Well-D, which was developed by our multidisciplinary team (eg, dietitians, nutrition professionals, and software engineers) [16,20]. Well-D was designed for participants to log consumed foods or dietary supplements and learn about adequate nutrient intake according to the Dietary Reference Intakes for Koreans 2015 and major foods contributing to key nutrients, including total energy, through personalized feedback. A detailed description of Well-D has been reported elsewhere [16].

In the intervention arm, participants were instructed to use Well-D for at least 14 days. Contact was minimal except when technical questions were raised. Since Well-D was implemented as a hybrid app, users who had either iOS or Android smartphones could freely access the app in a network environment. To facilitate app usage, dietitian staff sat with users and helped them register and log into the app. The users also reviewed all the menus in the app with the dietitian staff and were provided a Well-D manual. Users typed in their age, sex, weight, height, and physical activity level during registration. The app provides a database of more than 20,000 foods and recipe items. For foods and dietary supplements that are not available in the database, users could add new food data by typing in the item name and describing the item or adding a photo. The users could also create new recipe data by typing in the ingredients from a food list. Dietitians checked the items that users created and updated the recipe and the food and nutrient database. Based on users’ age, sex, BMI, physical activity level, and foods and supplements that they recorded, users received real-time feedback about daily total energy, carbohydrates, protein, total fat, sodium, saturated fat, fiber, sugar, calcium, vitamin C, riboflavin, and food groups on the diabetic exchange list. All of the data on the intake of foods, supplements, and nutrients were collected. We reviewed and downloaded the data from the admin page of the website.

In the paper-based diary group, participants were provided paper-based diaries and pamphlets. The paper-based diary was designed to record the date, time, name and amount of food and ingredients consumed, and the energy intake that participants roughly calculated. Each participant in the paper-based diary was provided a pamphlet that had tips about weight loss strategies and information on the website and URLs available for calculating the energy content of food items. We also provided instructions on how participants could set a proper energy intake goal via the paper-based diary and pamphlet, and participants wrote their energy intake goal for weight loss in the paper-based diary.

Our study design and results were presented according to the CONSORT-eHEALTH (Consolidated Standards of Reporting Trials of Electronic and Mobile Health Applications and Online Telehealth) guidelines [21].

#### Outcome Assessments

Weight, BMI, waist circumference, body fat mass, and skeletal muscle mass were measured as primary outcomes at baseline and after 6 weeks of intervention. Height was measured only at baseline. On the day before measurements, we sent text messages to inform participants to avoid large meals before the visit and to wear light clothing for the measurements. Height was measured twice to the nearest 0.1 cm without shoes using a digital stadiometer (Biospace Korea). Body weight was measured to the nearest 0.1 kg on the Inbody 720 (Biospace Korea) with participants wearing light clothing [22]. BMI was calculated as weight (kg) divided by the squared height (m^2^). Waist circumference was measured 1 inch above the umbilicus to the nearest 0.1 cm with a tape measure. For body composition, we assessed body fat mass and skeletal muscle mass using the Inbody 720 [22].

The participants’ diets were assessed using scheduled 24-hour recalls (24HRs) for 3 days including 1 day during a weekend. A dietitian conducted the 24HRs using the automated multiple-pass method (AMPM). AMPM uses five steps, including listing foods consumed the previous day, probing for forgotten foods, collecting the time of consumption, collecting descriptions about and amounts of each food, and final questions [23]. On the first day of the 3-day 24HR, participants visited the researcher’s office and completed the 24HRs. They completed the other 2 days of 24HRs over the phone. Three-day 24HRs were conducted at baseline and in week 6. We provided a booklet for a sample serving size of foods to all the participants in both groups to help estimate the amount of foods that they consumed. The amount of nutrients were calculated using databases sourced from the Diet Evaluation System by SAS 9.4 and Microsoft Excel 2013 software [24]. If the foods and dietary supplements were not available in the Diet Evaluation System database, we updated the additional food composition databases based on open-source food composition databases from the Ministry of Food and Drug Safety and the National Institute of Agricultural Sciences [25,26]. App and paper-based diary usage was measured by the number of days recorded from baseline to the endpoint visit. We considered it one recording day if users logged at least one food item.

Participants recorded self-reported physical activity using a South Korean version of the Global Physical Activity Questionnaire (GPAQ) [27]. We calculated metabolic equivalent of task (MET)-hours per week using the GPAQ analysis guideline [28]. Participants were advised to maintain their usual physical activity levels during the intervention.

### Statistical Analysis

We estimated the sample size to detect statistically significant differences in weight change between the app group and paper-based diary group using a similar previous 6-week trial [11]. By assuming a 0.9 kg difference between the two groups and a standard deviation of 1 kg, the calculated sample size with 80% power was 21 per group. Considering loss to follow-up, we recruited 25 participants per group.

All data were analyzed with intent-to-treat analysis. The differences in changes in anthropometric measures and nutrient intake between the app group and the paper-based diary group were analyzed by independent *t* tests for normally distributed data and Wilcoxon Mann-Whitney tests for skewed data. The differences between pre- and postintervention within each group were compared using paired *t* tests for normally distributed data and Wilcoxon signed-rank tests for skewed data. Data were transformed into normality, if necessary, using Box-Cox power transformations [29]. Carbohydrate, protein, fat, and saturated fat intakes were calculated as a percent of energy. We used a simple linear regression to evaluate the correlation between the number of days spent recording foods and weight change in each group.

There was one missing value for weight and two for other primary outcomes. The missing outcomes were carried forward from the baseline assessments. We also conducted a sensitivity analysis where we used per-protocol analysis by excluding those with missing outcomes.

## Results

Table 1 shows baseline characteristics by intervention arms. The mean age of participants was 26.0 years. The mean weight was 77.1 kg and the mean BMI was 26.7 kg/m^2^. The mean daily intake of energy was 2166.1 kcal/day. On average, % energy from carbohydrates, protein, and fat were 50.5%, 18.6%, and 30.9%, respectively. There were no significant differences in baseline characteristics between the app group and the paper-based diary group.

After randomization, one lactating participant was excluded (Figure 1). As a result, 33 men and 17 women aged 18-39 years participated in a 6-week RCT, and 32 men and 17 women completed the study. One participant in the paper-based diary group did not respond to final contact whereas another participant responded but could not attend a follow-up interview. This participant provided his weight and the number of days that he used the dietary diary. All participants completed 3-day 24HRs at baseline; 47 participants completed 3-day 24HRs and 1 participant in the app group completed 2 days of 24HRs during the 6-week intervention period.

**Figure 2:**
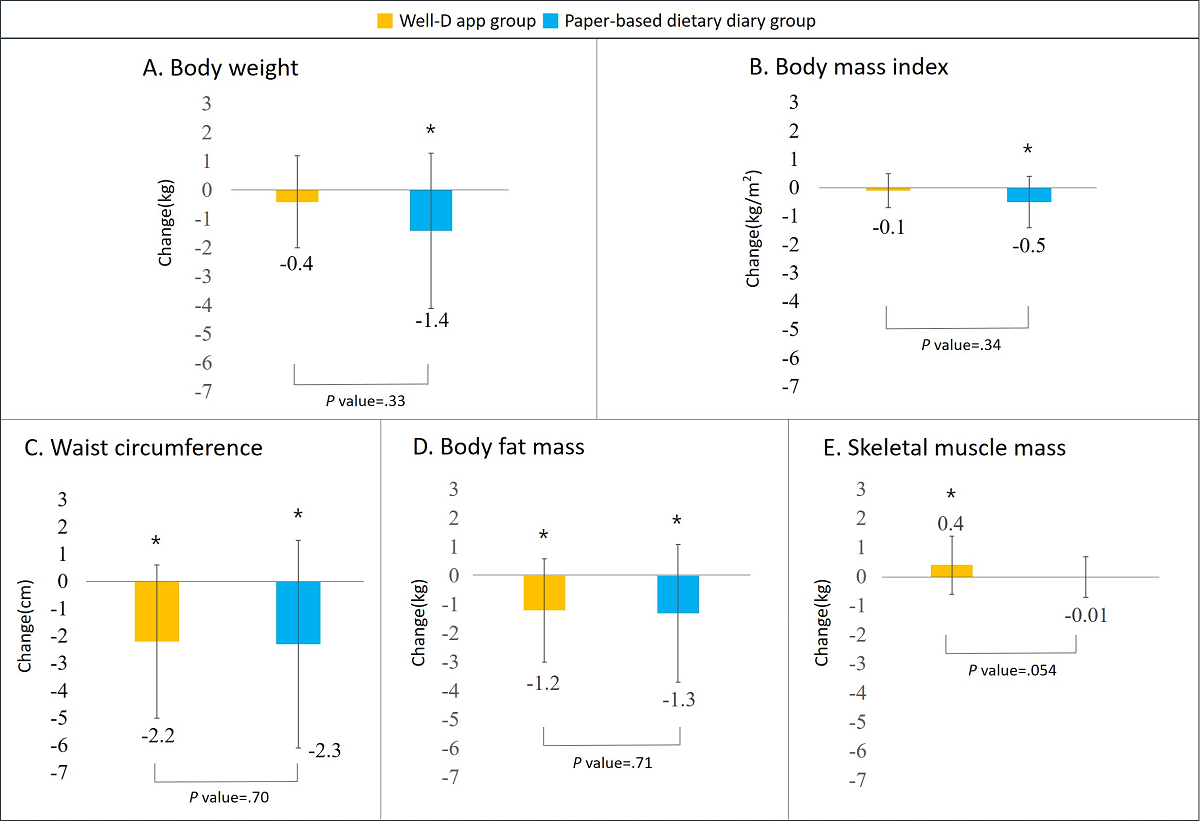
Differences in change between the app group and the paper-based diary group in terms of (A) body weight, (B) BMI, (C) waist circumference, (D) body fat mass, and (E) skeletal muscle mass. The asterisk denotes *P*<.05 (significant difference) for comparisons of anthropometric measures pre- to postintervention.

We found no statistically significant difference in change in body weight between the app group and the paper-based diary group (mean –0.4, SD 1.6 kg vs mean –1.4, SD 2.7 kg*;* *P*=.33) (Figure 2). Likewise, differences in changes in BMI, waist circumference, body fat mass, and skeletal muscle mass were not statistically significant between the two groups. Mean change and standard deviation between pre- and postintervention are presented in Table 2.

When we compared the preintervention anthropometric measures with the postintervention measures, significant decreases in body weight and BMI were observed in the paper-based diary group (*P*=.02 and .01, respectively), but not in the app group (*P*=.25 and .26, respectively). Waist circumference and body fat mass decreased significantly in both groups. The skeletal muscle mass significantly increased in the app group (*P*=.048). Overall, the results were similar in the sensitivity analysis where a per-protocol analysis was conducted by excluding those with missing anthropometric measures (Multimedia Appendix 1).

**Table 1:** Baseline characteristics of participants by intervention arms.

Characteristics		Total	App group	Paper-based diary group	*P* value^a^
**Sex,** **n (%)**					.77
	Male	33 (66)	16 (64)	17 (68)	
	Female	17 (34)	9 (36)	8 (32)	
Age (year), mean (SD)		26.0 (4.8)	26.5 (5.3)	25.6 (4.3)	.50
Weight (kg), mean (SD)		77.1 (11.5)	77.9 (12.9)	76.3 (10.2)	.62
BMI (kg/m^2^), mean (SD)		26.7 (2.7)	27.1 (3.0)	26.4 (2.5)	.54
Waist circumference (cm), mean (SD)		91.7 (9.3)	93.1 (9.6)	90.3 (8.9)	.25
Body fat mass (kg), mean (SD)		23.3 (6.3)	24.2 (5.6)	22.3 (6.9)	.29
Skeletal muscle mass (kg), mean (SD)		30.3 (6.1)	30.2 (6.5)	30.4 (5.7)	.91
Total physical activity (MET^b^-hours/week), mean (SD)		26.4 (25.6)	25.3 (23.4)	27.6 (28.1)	.87
**Energy (kcal/day), mean (SD)**		2166.1 (546.5)	2270.3 (522.1)	2061.9 (560.8)	.18
	Carbohydrate (% energy/day)	50.5 (6.7)	48.8 (7.8)	52.1 (5.1)	.11
	Protein (% energy/day)	18.6 (4.0)	19.3 (4.2)	17.8 (3.6)	.34
	Fat (% energy/day)	30.9 (5.7)	31.7 (6.7)	30.0 (4.4)	.34
Total dietary fiber (g/day), mean (SD)		17.0 (6.1)	16.7 (5.9)	17.4 (6.4)	.69
Calcium (mg/day), mean (SD)		530.8 (237.9)	512.8 (185.3)	548.8 (283.8)	.74
Sodium (mg/day), mean (SD)		4021.7 (1157.2)	4110.6 (1124.0)	3932.7 (1205.8)	>.99

^a^Chi-square tests were used for categorical variables, and independent *t* tests or Wilcoxon Mann-Whitney tests were used for continuous variables.

^b^MET: metabolic equivalent task.

**Table 2:** Differences in anthropometric measures between the app group and the paper-based diary group (intent-to-treat analysis).

Measure	App group (n=25), mean (SD)				Paper-based diary group (n=25), mean (SD)					*P* value^a^
	Baseline	6 weeks	Change^b^	*P* value^c^	Baseline	6 weeks	Change^b^	*P* value^c^		
Weight (kg)	78.0 (12.9)	77.6 (13.0)	–0.4 (1.6)	.25	76.3 (10.2)	75.0 (9.3)	–1.4 (2.7)	.02	.33	
BMI (kg/m^2^)	27.1 (3.0)	26.9 (3.0)	–0.1 (0.6)	.26	26.4 (2.5)	25.9 (2.2)	–0.5 (0.9)	.01	.34	
Waist circumference (cm)	93.1 (9.6)	90.9 (9.2)	–2.2 (2.8)	<.001	90.3 (9.0)	88.1 (7.1)	–2.3 (3.8)	.004	.70	
Body fat mass (kg)	24.2 (5.6)	23.0 (6.1)	–1.2 (1.8)	.004	22.3 (6.9)	21.0 (5.9)	–1.3 (2.4)	.01	.71	
Skeletal muscle mass (kg)	30.2 (6.5)	30.6 (6.6)	0.4 (1.0)	.048	30.4 (5.7)	30.4 (5.8)	–0.01 (0.7)	.48	.054	

^a^Independent *t* tests or Wilcoxon Mann-Whitney tests were used to assess the differences in percent changes in anthropometric measures between the app group and the paper-based diary group.

^b^Changes were calculated as postintervention anthropometric measures minus preintervention anthropometric measures.

^c^Paired *t* tests or Wilcoxon signed-rank tests were used to assess the differences in percent changes in anthropometric measures pre- to postintervention.

Differences in changes in nutrient intake between the app group and the paper-based diary group were not statistically significant (Table 3). When we compared nutrient intake assessed from preintervention 24HRs with postintervention 24HRs, we observed significant reductions in cholesterol, calcium, phosphorus, and potassium only in the paper-based diary group (all *P*s<.05). When we compared nutrient intake levels from the Well-D app with those from the 24-hour recalls (24HR) among 25 participants in the intervention arm, we found moderate-to-high correlations (from 0.43 for thiamine to 0.71 for iron) (Table 4).

**Figure 3:**
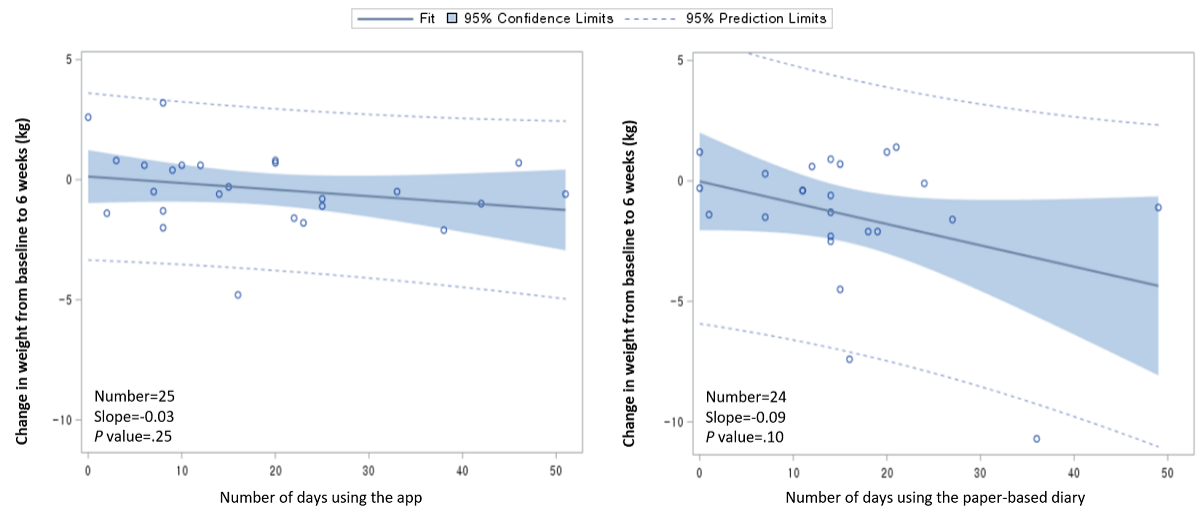
Weight loss by the number of days of dietary recording in the app group (left) and the paper-based diary group (right).

Over the 6-week intervention period, differences in the number of days recorded was not significant between the app group and the paper-based diary group (mean 18.5, SD 14.1 vs mean 15.5, SD 10.1, respectively; *P*=.67). We examined whether the degree of weight loss was associated with the number of days spent using the app or the paper-based diary (Figure 3). The change in body weight from pre- to postintervention tended to increase according to the number of days participants recorded an entry. However, the beta coefficient was not statistically significant.

When we counted the number of participants who recorded food items in each week, we found higher proportions of recording in week 1 and week 2 in both groups than in later weeks and a higher proportion of recording, in general, in the app group than in the paper-based diary group. In all, 51.43% (12.86 on average per day for 7 days) of participants recorded food items in the app group and 38.10% (8 on average per day) of participants recorded food items in the paper-based diary group in week 1, but the proportions decreased to 36.00% (9 on average per day) in week 5 in the app group and 32.65% (6.86 on average per day) in the paper-based dairy group (Table 5).

**Table 3:** Differences in changes in nutrient intake assessed from 24-hour recalls between the app group and the paper-based diary group (intent-to-treat analysis).

Characteristic	App group (n=25), mean (SD)			Paper-based diary group (n=25), mean (SD)				*P* value^a^	
	Baseline	6 weeks	*P* value^b^		Baseline	6 weeks	*P* value^b^		
Energy (kcal/day)	2269.7 (522.8)	1983.5 (365.3)	.04		2061.9 (560.8	1780.6 (571.0)	.06		.98
Carbohydrate (% energy/day)	48.8 (7.8)	48.9 (8.5)	.95		52.2 (5.1)	49.8 (8.3)	.20		.34
Protein (% energy/day)	19.3 (4.2)	19.7 (4.4)	.68		17.8 (3.6)	17.4 (3.5)	.63		.60
Fat (% energy/day)	31.9 (7.0)	31.4 (7.4)	.70		30.0 (4.4)	32.8 (7.6)	.13		.18
Saturated fat (% energy/day)	12.1 (6.2)	12.2 (6.5)	.83		10.4 (2.3)	10.1 (2.4)	.50		.76
Total dietary fiber (g/day)	16.7 (5.9)	15.3 (5.1)	.42		17.4 (6.4)	15.0 (5.7)	.10		.64
Cholesterol (mg/day)	371.9 (122.7)	363.8 (160.3)	.70		363.0 (146.2)	289.0 (100.7)	.04		.35
Calcium (mg/day)	512.8 (185.3)	525.2 (312.4)	.57		548.8 (283.8)	438.9 (241.9)	.01		.23
Phosphorus (mg/day)	1076.5 (300.2)	1041.9 (297.1)	.63		1067.6 (281.1)	878.1 (268.6)	.01		.14
Iron (mg/day)	16.8 (12.3)	14.5 (7.9)	.43		19.7 (32.3)	17.9 (32.4)	.05		.30
Sodium (mg/day)	4110.6 (1124)	3833.4 (1176.3)	.23		3932.7 (1205.8)	3531.9 (1596.0)	.21		.77
Potassium (mg/day)	2254.4 (562.3)	2318.1 (743.5)	.77		2325.9 (658.3)	2015.7 (587.8)	.01		.07
Vitamin A (μg RE^c^/day)	742.0 (937.1)	785.2 (1087.6)	.83		588.4 (716.9)	574.1 (580.0)	.84		.55
Thiamine (mg/day)	2.7 (5.1)	2.9 (5.9)	.22		6.3 (21.5)	1.6 (1.8)	.05		.42
Riboflavin (mg/day)	2.9 (5.3)	2.9 (5.8)	.78		5.9 (21.1)	1.5 (1.3)	.09		.35
Niacin (mg/day)	25.7 (28.7)	24.3 (30.8)	.76		25.2 (28.7)	18 (10.2)	.26		.54
Vitamin C (mg/day)	276.4 (338.0)	201.7 (314.2)	.07		127.3 (120.0)	154.7 (281.8)	.42		.46

^a^Independent *t* tests and Wilcoxon Mann-Whitney tests were used to assess group differences.

^b^Paired *t* tests or Wilcoxon signed-rank tests were used to assess differences in percent changes in nutrient intake pre- to postintervention.

^c^RE: retinol equivalents.

**Table 4:** A comparison of nutrient intake levels from the Well-D app with those from the 24-hour recalls (n=25).

Nutrient^a^	Correlation coefficient	*P* value^b^
Energy (kcal/day)	0.68	<.01
Carbohydrate (% energy/day)	0.55	<.01
Protein (% energy/day)	0.60	<.01
Fat (% energy/day)	0.57	<.01
Saturated fat (% energy/day)	0.66	<.001
Total dietary fiber (g/day)	0.51	.01
Cholesterol (mg/day)	0.47	.02
Calcium (mg/day)	0.49	.01
Phosphorus (mg/day)	0.47	.02
Iron (mg/day)	0.71	<.001
Sodium (mg/day)	0.56	<.01
Potassium (mg/day)	0.56	<.01
Vitamin A (μg RE/day)	0.54	.01
Thiamine (mg/day)	0.43	.03
Riboflavin (mg/day)	0.50	.01
Niacin (mg/day)	0.70	<.001
Vitamin C (mg/day)	0.66	<.001

^a^The residual method was used to adjust for nutrient intake.

^b^Either Pearson correlation or Spearman correlation was used.

**Table 5:** Mean number (percentage) of days participants spent recording food items for each week in the app group and in the paper-based diary group.

Week	App group (n=25), mean (%)	Paper-diary group (n=21)^a^, mean (%)
Week 1	12.86 (51.43)	8.00 (38.10)
Week 2	11.43 (45.71)	8.14 (38.78)
Week 3	9.57 (38.29)	8.57 (40.82)
Week 4	9.00 (36.00)	6.86 (32.65)
Week 5	9.00 (36.00)	6.86 (32.65)
Week 6	10.00 (40.00)	4.29 (20.41)

^a^Out of 25, one was lost to follow-up. Three did not provide information on dates recorded, but provided the number of days recorded.

## Discussion

### Principal Findings

We conducted an RCT on weight loss in young adults with a BMI ≥23 kg/m^2^ to compare the effectiveness of a mobile dietary self-monitoring app versus a paper-based diary. In our study, 66% of participants were men, which reflects the sex ratio of overweight individuals in Korea [4]. The mean age was 26 years since we recruited participants in college. The average total energy intake was 2166.1 kcal/day with an average of 50.5% of energy derived from carbohydrates, 30.9% energy from fat, and 18.6% from protein. The average sodium intake was 4021.7 mg/day.

In summary, we did not find significant differences in changes in body weight, BMI, waist circumference, body fat mass, or skeletal muscle mass between the app group and the paper-based diary group. Additionally, the changes in nutrient intake were not different between the two groups. However, when we compared anthropometric changes from pre- to postintervention, we found reductions in weight and BMI in the paper-based group and reductions in waist circumference and body fat mass in both groups. Skeletal muscle mass increased slightly in the app group. We also found decreases in total energy intake in both groups and as well as decreases in intake of cholesterol, calcium, phosphorus, and potassium in the paper-based diary group, but not in the app group. When we compared nutrient intake from the Well-D app with 24HRs, we found modest-to-high correlations, suggesting potential use of Well-D for dietary assessment.

### Comparison With Previous Work

Previous studies found that the effectiveness of mHealth technologies was similar to the paper-based diary [11-13]. A 24-month randomized trial with 210 overweight or obese adults compared the effectiveness of a PDA for self-monitoring diet, a PDA with daily feedback, and a paper diary. There were no differences in changes in percentage weight over time among the three groups, but a significant weight change over time was found only for the PDA with feedback group [12]. The mean percentages of weight change from baseline to 24 months were –1.94% for the paper diary (not significant), –1.38% for the PDA (not significant), and –2.32% for the PDA + feedback (significant). The study suggested that daily feedback enhanced adherence to self-monitoring and the effectiveness of weight loss. Another randomized trial compared weight loss between the Lose it mobile app and a traditional paper-and-pencil method among 47 overweight or obese adults during an 8-week intervention. Weight was reduced over the course of the study in both groups, but there was no difference in weight change between groups. This study showed that the number of days spent recording diet was significantly higher in the app group than the paper-and-pencil group [13]. The 6-month trial of the My Meal Mate app showed that, among 128 overweight participants, weight change over time was significantly greater in the smartphone group than in the website group, but there was no difference in the smartphone group compared to the diary group [11]. In the intent-to-treat analysis of that study, mean weight changes from baseline to 6 months were –4.6 kg in the smartphone app group, –2.9 kg in the diary group, and –1.3 kg in the website group. The smartphone-based behavioral obesity treatment (SMART) randomized clinical trial of 276 adults with overweight or obesity over 18 months recently published findings of similar weight loss across the three groups: group-based treatment with meetings (GROUP), SMART-based treatment, and a control condition with paper diaries (CONTROL) [30]. In that study, although retention was higher in both GROUP and SMART groups than the CONTROL group, weight changes did not differ at the 6-month, 12-month, or 18-month assessment. Consistent with a few previous studies, we found that changes in anthropometric measures were not different between the app group and paper-based diary group.

There have been only a few studies that have supported the effectiveness of an app to improve users’ diet compared to a paper-based method [14,15]. A 4-week pilot intervention study with the MyFitnessPal app among 30 healthy adults showed that the app users who received feedback on sodium intake significantly decreased their urinary sodium compared to the paper-based journal group [15]. In another two-phase crossover intervention study, 34 adolescents recorded food intake using a paper diary and the FoodWiz2 app during each 4-week intervention period [14]. The app phase showed a significant reduction in the consumption of chocolate snacks and fizzy drinks in comparison to the paper diary phase [14]. In our study, when we compared nutrient intake between the app group and the paper-based diary group, we did not find significant differences. However, we found decreases in total energy intake in both app and paper-based diary groups.

Several studies conducted in South Korea reported potential weight change when using mHealth tools. A longitudinal study with a median follow-up of 275 days investigated the effectiveness of the Noom Coach app on weight reduction among 35,921 South Korean adults with a BMI ≥23 kg/m^2^, who recorded dietary data two or more times a month for 6 consecutive months [31]. That study found that 22.7% of app users reduced their weight by more than 10% compared to the baseline weight. Similarly, a weight loss intervention study of 104 Korean adults aged 20-60 years with a BMI ≥23 kg/m^2^ showed that the use of the Noom Coach app with daily dietary coaching resulted in a significant weight change of –7.5% after a 15-week intervention period compared to baseline [32].

Additionally, a randomized trial that compared weight loss according to the frequency of usage of the My Meal Mate app found that participants in the highest frequency-of-use category lost an average of 6.4 kg more than those in the lowest frequency-of-use category during the 6-month intervention period [33]. A cohort study showed that more app-based recordings were associated with greater weight loss [31]. In the Cell Phone Intervention For You (CITY) trial, increasing app use per day was associated with increasing weight loss [34]. In our study, we observed a tendency for greater weight loss as the number of recordings increased, albeit without statistical significance.

### Strengths and Limitations

Our study was a randomized, parallel trial with a high follow-up rate. Because we developed the app, we had full access to the data. We also assessed participants’ dietary information using two 3-day 24HRs, and therefore we were able to compare nutrient intake between the two groups. However, our study had several limitations. Because the population was composed of young adults, the results may not be generalizable to children or older people. Since we included participants with a BMI ≥23 kg/m^2^, the magnitude of weight change during the 6-week intervention period may not be large enough to see differences. Furthermore, the sample size was small and the study period was relatively short. Well-D did not have an exercise tracking function, and we were not able to track participants’ exercise levels. However, we asked participants to maintain their usual physical activity levels. When we examined their usual exercise levels at baseline and at postintervention, we found no significant differences.

### Conclusions

We conducted an RCT to evaluate the effectiveness of a mobile dietary self-monitoring app for weight loss versus a paper-based diary. We found that participants reduced their energy intake, waist circumference, and body fat mass in both groups. However, we found no difference in changes between the app group and paper-based diary group. Our study suggests that both the smartphone app and the paper-based diary method may exhibit similar effectiveness for short-term body fat loss. Our findings may contribute to understanding and implementing mHealth interventions in health care services. Further prospective or intervention studies with larger sample sizes and long-term follow-up are warranted to explore the effectiveness of mHealth tools for the management of common chronic diseases in Korea.

## Appendix

Multimedia Appendix 1 Differences in anthropometric measures between the app group and the paper-based diary group (per-protocol analysis).

Multimedia Appendix 2 CONSORT eHEALTH (V1.6.1).

## Abbreviations

24HR: 24-hour recall
AMPM: automated multiple-pass method
CITY: Cell Phone Intervention For You
CONSORT-eHEALTH: Consolidated Standards of Reporting Trials of Electronic and Mobile Health Applications and Online Telehealth
GPAQ: Global Physical Activity Questionnaire
ICT: information and communications technology
MET: metabolic equivalent of task
mHealth: mobile health
NCD: noncommunicable disease
PDA: Personal Digital Assistant
RCT: randomized controlled trial
SMART: smartphone-based behavioral obesity treatment
WHO: World Health Organization
