# Supplementary material for: Use of a Smartphone App for Weight Loss Versus a Paper-Based Dietary Diary in Overweight Adults: Randomized Controlled Trial
Source: JMIR Mhealth Uhealth. 2020 Jul 31;8(7):e14013. doi: 10.2196/14013 (PMC7428925; doi:10.2196/14013)
Supplement: Multimedia Appendix 1 [file mhealth_v8i7e14013_app1.docx]

**Multimedia Appendix 1**. Differences in anthropometric measures between the app group and the paper-based diary group (per-protocol analysis)

|  | App group (n=25), mean (SD) | | | | Paper-based diary group (n=25), mean (SD) | | | | *P* value^a^ |
| --- | --- | --- | --- | --- | --- | --- | --- | --- | --- |
|  | Baseline | 6 wk | Change^b^ | *P* value^c^ | Baseline | 6 wk | Change^b^ | *P* value^c^ |  |
| Weight (kg) | 78.0 (12.9) | 77.6 (13.0) | -0.4 (1.6) | .25 | 76.3 (10.2) | 75.0 (9.3) | -1.4 (2.8) | .02 | .29 |
| BMI (kg/m^2^) | 27.1 (3.0) | 26.9 (3.0) | -0.1 (0.6) | .26 | 26.4 (2.5) | 25.9 (2.2) | -0.5 (0.9) | .01 | .30 |
| Waist circumference (cm) | 93.1 (9.6) | 90.9 (9.2) | -2.2 (2.8) | <.001 | 90.3 (9.0) | 88.1 (7.1) | -2.3 (3.9) | .004 | .93 |
| Body fat mass (kg) | 24.2 (5.6) | 23.0 (6.1) | -1.2 (1.8) | .004 | 22.3 (6.9) | 21.0 (5.9) | -1.4 (2.5) | .01 | >.99 |
| Skeletal muscle mass (kg) | 30.2 (6.5) | 30.6 (6.6) | 0.4 (1.0) | .048 | 30.4 (5.7) | 30.4 (5.8) | -0.01 (0.8) | .94 | .10 |

^a^ Independent t-tests or Wilcoxon Mann-Whitney tests were used to assess the differences in percent changes in anthropometric measures between the app group and the paper diary group.

^b^ Changes were calculated as postintervention anthropometric measures minus pre-intervention anthropometric measures.

^c^ Paired t-tests or Wilcoxon signed-rank tests were used to assess the differences in percent changes in anthropometric measures pre- to postintervention
